# The Essential Vulnerability of Human Cardiac Myocytes to SARS-CoV-2∗

**DOI:** 10.1016/j.jacbts.2021.02.010

**Published:** 2021-04-27

**Authors:** Mitali Das, Michael R. Bristow, Mina K. Chung

**Affiliations:** a Department of Cardiovascular Medicine, Heart, Vascular and Thoracic Institute, Cleveland Clinic, Cleveland Clinic Lerner College of Medicine of Case Western Reserve, University School of Medicine, Cleveland, Ohio, USA; b Department of Medicine, Division of Cardiology, Cardiovascular Institute, University of Colorado, Denver/Anschutz Medical Campus, Aurora, Colorado, USA

**Keywords:** cardiomyocytes, COVID-19, myocarditis, SARS-CoV-2

**Figure:**
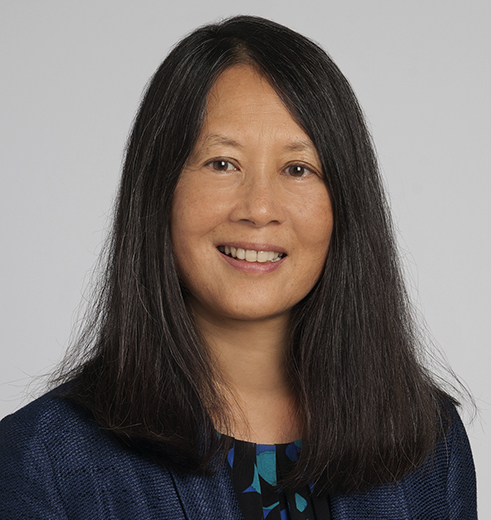

As the coronavirus disease-2019 (COVID-19) pandemic unfolded, cardiovascular vulnerability to the effects of severe acute respiratory syndrome-coronavirus-2 (SARS-CoV-2) infection became increasingly evident. Elevated cardiac biomarkers that indicated evidence of cardiac injury were elevated in 8% to 62% of hospitalized COVID-19 patients and were associated with more severe disease and higher mortality. Patients with cardiovascular disease and risk factors, such as older age, obesity, and hypertension, appeared to be particularly susceptible to cardiac injury and poor outcomes. Moreover, evidence of myocarditis and/or myocardial scarring has been reported in cardiac magnetic resonance imaging studies in patients who have recovered from COVID-19, including collegiate athletes who had no or minimal symptoms during initial infection.

Despite such indications of cardiac injury, demonstrations of direct viral infection of myocardial cells from autopsies or from endomyocardial biopsies have been sparse. In a review of 277 cardiac autopsies across 22 studies, only 20 cases (7.2%) of myocarditis were reported (1), and although reverse transcription polymerase chain reaction has detected viral RNA in the heart, it has been unclear whether that finding represented infection of cardiac cells or circulating viral particles. On this background, multiple laboratories have been investigating the susceptibility of human myocardial cells and, particularly, cardiac myocytes to SARS-CoV-2 infection and subsequent tissue damage. By using a diverse array of corroborating cellular, functional, pharmacologic, and transcriptomic approaches in human origin cells and tissue, the study by Bailey et al. (2) helps to settle ambiguities over whether SARS-CoV-2 can infect the heart directly and cardiac myocytes specifically, and whether the ensuing tissue injury and cell damage are secondary to direct cytopathic or inflammatory mechanisms. In the clinical pathology portion of their report, myocardial tissue is examined from 4 autopsy or endomyocardial biopsy COVID-19 patients with clinical myocarditis (2). In all 4 autopsies, cardiac myocyte infection was demonstrated by identifying intracellular viral particles, budding on electron microscopy, cell death, and macrophage infiltration associated with areas of myocyte cell death. This represents the largest number of patients reported with COVID-19-associated myocarditis who had myocardial histopathologic examinations and identification of SARS-CoV-2 in cardiac myocytes.

Cardiac tropism in COVID-19 infection is expected, as the main host cell receptor for SARS-CoV-2 spike protein is the highly myocardium-expressed angiotensin-converting enzyme 2 (ACE2), and ventricular myocardium (3), including cardiac myocytes (4), express all the additional machinery required for virus-host cell membrane fusion and cell entry. Single nuclei RNA sequencing (snRNAseq) studies have reported robust expression of ACE2 in cardiac vascular pericytes with lower expression in ventricular cardiac myocytes and fibroblasts (4). ACE2 expression is upregulated in left ventricle remodeling (3,4) where snRNAseq studies showed myocyte-specific upregulation. Therefore, these studies predict enhanced vulnerability of the heart to SARS-CoV-2 infection, which would be expected to readily bind to cardiac myocytes, particularly in the presence of myocardial hypertrophy, as well as to pericytes, potentially contributing to the vascular thrombotic or endotheliitis complications of SARS-CoV-2.

The translational component of the study by Bailey et al. (2) is a methodological tour de force that uses human-induced pluripotent stem cells (hiPSC)-derived cardiac myocytes and endothelial cells; cultured human cardiac endothelial cells; fibroblasts, macrophages, isolated human ventricular and atrial cardiac myocytes; and engineered human heart tissue (EHT) to model SARS-CoV-2 infection. These experiments elegantly demonstrated cardiac myocyte-specific effects on susceptibility to infection, cytopathic effects, and changes in gene expression. Using combined cultures of cardiomyocyte, fibroblast, and macrophages, the investigators confirmed cell-specific delineation of cardiac myocytes as targets for SARS-CoV-2 (2). They demonstrated that hiPSC-derived cardiac myocytes were easily and robustly infected but that other cells, including undifferentiated hiPSCs, were not susceptible. Furthermore, this highly selective cellular tropism correlated with expression of ACE2, which was not expressed in fibroblasts, endothelial cells, or macrophages. Infection in cardiac myocytes was blocked by an ACE2 antibody, remdesivir or a cathepsin inhibitor, but not by a serine protease inhibitor.

Prior studies have also used hiPSC-derived cardiac myocytes to investigate SARS-CoV-2 infection in cardiac myocytes and also reported relative ease of infection (5,6); dependence on ACE2 (5,6); similar cytopathic effects (5,6), including cell death and apoptosis (5); cathepsin involvement in cell entry (6); and comparable patterns of altered gene expression (5). The translational value of the study by Bailey et al. (2) is its use of a broad spectrum of models incorporating multiple controls of nonmyocyte myocardial cells. In addition, the clinical pathology material not only provided a histopathologic comparison for their translational studies but also demonstrated that SARS-CoV-2 can be found in the hearts of clinically infected patients if one looks for it in patients with clinical evidence of myocardial involvement.

Data from the studies by Bailey et al. (2) and Bojkova et al. (6) suggest that cardiac myocytes may exhibit cell entry differences compared to lung epithelial cells (7). Cardiac myocytes demonstrated infection dependence for cathepsins, which can process spike protein to enable cell fusion and incorporation of virus into endosomes (5,6). In contrast, in lung epithelial cells, membrane priming and fusion following binding to ACE2 is mediated by the transmembrane serine protease (TMPRSS2) (7). The explanation for this difference may be that human ventricular myocardium (3), cardiac myocytes (4), and hiPSCs (2,6) contain very low or undetectable levels of TMPRSS2.

An important contribution by the study by Bailey et al. (2) is the observation of decreased ACE2 expression after hiPSC cardiac myocyte infection. Although this had been reported for the original SARS-CoV in autopsies from infected, histologically damaged human hearts, until recently (8) there have been no data in human myocardium from COVID-19 patients. The single previous report of decreased levels of ACE2 in patients with a SARs-CoV-2 was in subjects that did not have evidence of myocarditis and had died of a variety of causes not directly related to apparent SARS-CoV-2 cardiac involvement (8), and more work needs to be done investigating that issue. Nevertheless, in hiPSC cardiac myocytes (2), the finding of infection-related down-regulation in ACE2 could be related to receptor internalization or shedding. This is an important issue because of the essential function of ACE2 in the heart with its role of regulating angiotensin II levels and generating the counter-regulatory peptide angiotensin (1, 2, 3, 4, 5, 6, 7). However, no expression levels of the *ACE* gene or the ACE2-to-ACE ratio were reported (2), so the significance of this finding as well as its pharmacologic specificity are uncertain.

Viral infection negatively impacted the expression of genes associated with the critical cardiac myocyte functions of contractility and energetics, including downregulation of those involved in calcium handling, sarcomere function, glucose metabolism, the Tricarboxylic acid cycle and oxidative phosphorylation. These changes would clearly explain the decrease in contractile function observed in infected EHT preparations. In contrast, mediators of the innate immune response, cytokines and inflammation-response genes were upregulated in these models, showing that inflammation was indeed a direct effect of SARS-CoV-2 infection of cardiomyocytes or EHTs. Interestingly, although macrophages and fibroblasts were not susceptible to infection, they also contributed to the repertoire of chemokines and cytokines expressed by the infected EHTs. Preventing viral entry through an ACE2-neutralizing antibody or viral replication by remdesivir alleviated the inflammatory gene expression in EHTs, showing that viral infection in the EHT model can set up a local inflammatory cascade. Although both inhibition of type I interferon activity (by inhibition of TBK1) and viral replication by remdesivir preserved contractile protein mRNA expression, it was only remdesivir that was able to prevent infection mediated sarcomere disassembly. By showing that infection in these cardiac myocyte and EHT models triggered immune responses, these studies demonstrated that myocarditis can be induced by direct SARS-CoV-2 viral infection and is not merely a secondary effect of systemic inflammation. However, when the interferon pathway immune response to viral RNA sensing was inhibited by TBK1 the inflammatory signature was reduced, whereas sarcomere breakdown and cell death were not. These observations suggest the major mechanism for cardiac myocyte injury from SARS-Co-V2 is the direct cytopathic effect of cellular infection and not the generated immune response.

These studies identify cardiac myocytes as important components of myocardial injury and the inflammatory reaction during SARS-CoV-2 infection but also raise some questions. There remains a dichotomy between studies that do not find significant myocardial injury on autopsy and those that report a relatively high incidence of cardiac troponin elevations in clinical studies of COVID-19 inpatients. Transient reversible myocardial injury is a possible explanation for this discrepancy. It is also unclear whether signaling from cardiac myocytes or innate immune signaling from damage- or pathogen-associated molecular patterns triggers downstream effects in other cell lineages such as macrophages or fibroblasts that leads to cross-talk between cell types and the upregulated immune and cytokine responses observed in these studies. Unlike an snRNAseq-based investigation that detected mRNA expression in ventricular fibroblasts (4), the study by Bailey et al. (2) did not detect significant expression of the *ACE2* gene in various types of fibroblasts including those of atrial origin. This discrepancy suggests chamber specificity of *ACE2* expression or a methodologic issue leading to lower sensitivity of fibroblast *ACE2* mRNA detection in the experiments conducted by Bailey et al. (2). The role of pericytes in SARS-CoV-2 infection remains to be clarified but is likely important given the robust expression of *ACE2* in pericytes, an abundant type of cell in the heart. Endothelial cells have been reported to have very low expression of *ACE2* in the heart (4), but endothelial cell-pericyte cross-talk is important for maintaining microvascular, coagulation, and immune homeostasis. Pericytes surround capillaries and small vessels and may lay in close contact to cardiac myocytes as well (9). The high level of expression of *ACE2* on pericytes suggests a role in maintaining the balance of the renin-angiotensin system. Whether pericyte-cardiomyocyte cross-talk impacts SARS-CoV-2 infection of cardiac myocytes is unknown. Such mechanisms may include competitive inhibition of SARS-CoV-2 binding to cardiomyocyte *ACE2* by *ACE2* shed from nearby pericytes or alterations in angiotensin II balance. Moreover, the decrease in *ACE2* expression with viral infection raises the question of whether agents that could increase *ACE2* expression after settling of viral load may improve cardiac status once the virus has been cleared or inhibited by antiviral therapy (3).

The authors are to be commended for using hiPSC cardiac myocytes and other myocardial cell models to gain important mechanistic insights as to how SARS-CoV-2 infects the heart (2). However, there are some limitations to the study. hiPSC cardiac myocytes are not adult cells, and their ease of infection, cytopathic effects, and especially effects on gene expression may differ from those of myocytes in the intact human heart. The role of pericytes in cardiac infection with SARS-CoV-2 remains to be established. These cells can hopefully be added to future hiPSC-derived models. Whether the high *ACE2* expression in pericytes contributes to endothelial dysfunction or thrombosis remains uncertain but in conjunction with upregulated *ACE2* expression in failing hearts may contribute to multilevel risk to the heart during COVID-19 infection. However, from the standpoint of the business end of the heart, maintenance of contractile function through myocyte viability, this study contributes to the understanding of how the functionally essential enzyme ACE2 also exists as an Achilles heel, creating vulnerability to a potentially lethal virus.

## Funding Support And Author Disclosures

Supported by 810959 AHA Covid-19 Rapid Response Research Coordinating Center grant (to Dr. Chung); by AHA Covid-19 Rapid Response Research grants 811960 (to Dr. Bristow) and 814633 (to Dr. Chung); 16SFRN31420008 AHA Strategically Focused Heart Failure Research Network grant (to Dr. Bristow); and by U.S. National Institutes of Health/National Heart, Lung, and Blood Institute grant R01HL111314.The authors have reported that they have no relationships relevant to the contents of this paper to disclose.
